# Regulation of the FOXO3a/Bim signaling pathway by 5,7-dihydroxy-8-nitrochrysin in MDA-MB-453 breast cancer cells

**DOI:** 10.3892/ol.2012.1077

**Published:** 2012-12-14

**Authors:** XIAO-CHUN ZHAO, XIAO-CHENG CAO, FEI LIU, MEI-FANG QUAN, KAI-QUN REN, JIAN-GUO CAO

**Affiliations:** 1 Department of Oncology, First Affiliated Hospital of University of South China, Hengyang 421001;; 2 Laboratory of Medicine Engineering, Medical College, Hunan Normal University, Changsha 410013, P.R. China

**Keywords:** breast cancer, chrysin, 5,7-dihydroxy-8-nitrochrysin, Akt, forkhead box O3a, Bim

## Abstract

We previously demonstrated that 5,7-dihydroxy-8-nitrochrysin (NOC), a novel synthetic chrysin analog, preferentially inhibits HER-2/neu-overexpressing MDA-MB-453 breast cancer cell growth by inducing apoptosis; however, the precise molecular mechanism was unclear. In this study, we demonstrated that NOC significantly induces apoptosis of MDA-MB-453 cells and that this is primarily mediated through a mitochondrial death cascade. This was presented as a loss of mitochondrial membrane potential, release of cytochrome *c* and activation of caspase-9. NOC induces a significant increase in levels of the BH3-only protein Bim. Small interfering RNA-mediated knockdown of Bim markedly attenuated NOC-induced apoptosis. An upstream transcriptional regulator of Bim, forkhead box O3a transcription factor (FOXO3a), experienced a decrease in phosphorylation and nuclear translocation. Silencing of FOXO3a resulted in a marked attenuation in the expression of Bim, as well as protection against NOC-mediated apoptosis. Furthermore, NOC-induced activation and nuclear localization of FOXO3a was associated with reduced levels of Akt phosphorylation. These results suggest that NOC induces apoptosis in MDA-MB-453 human breast cancer cells via caspase activation and modulation of the Akt/FOXO3a pathway.

## Introduction

Breast cancer continues to be a leading worldwide cause of cancer-related mortality among females, despite significant advances in screening techniques that lead to early detection of the disease (1). The incidence of breast cancer is lower in Asia than in Western countries (2). This may be attributable to Asian diets that are rich in flavonoid-containing plants, which are thought to be anti-tumorigenic. Chrysin (5,7-dihydroxyflavone, ChR), a natural flavonoid present in daily diets, possesses the ability to inhibit growth and induce apoptosis in a variety of cancer cells, including cervical cancer (3), leukemia (4,5), colon carcinoma (6), esophageal adenocarcinoma (7) and lung adenocarcinoma (8). Poor oral bioavailability has been a major limitation for the successful use of dietary flavonoids as cancer chemotherapeutic agents (9,10).

In order to improve the biological activities of ChR, we synthesized 5,7-dihydroxy-8-nitrochrysin (NOC) (11). We previously identified that NOC inhibits proliferation of the colon cancer cell line, HT-29 and the gastric cancer cell line, SGC-7901 to a greater extent than ChR (11). Additionally, we demonstrated that NOC induces breast cancer cell apoptosis by generation of reactive oxygen species (ROS) and Akt dephosphorylation (12). However, the link between Akt dephosphorylation and apoptosis induction in breast cancer cells remains to be elucidated.

Forkhead box O3a transcription factor (FOXO3a) is a transcription factor that functions downstream in the Akt signaling pathway and it is an important regulator of cell death (13). A number of anticancer drugs, including doxorubicin and paclitaxel, induce apoptosis through oxidative stress, which enhances FOXO3a activity by stimulating FOXO3a dephosphorylation and nuclear translocation (14). This causes overexpression of FOXO3a-responsive genes, including Bim, p27 and p21 (15). Therefore, regulation of FOXO3a factors by the Akt pathway is receiving increasing attention in cancer research.

In this study, we demonstrated that NOC significantly induced apoptosis of the breast cancer cell line MDA-MB-453 and the underlying molecular mechanisms are associated with regulation of the Akt/FOXO3a signaling pathway.

## Materials and methods

### Cell line and cell culture

The human breast cancer cell line MDA-MB-453 (ER negative, HER-2/neu-overexpressing) was grown in Dulbecco’s modified Eagle’s medium (DMEM; Invitrogen Life Technologies, Carlsbad, CA, USA) supplemented with 10% fetal bovine serum (Invitrogen Life Technologies), 100 U/ml penicillin and 100 U/ml streptomycin in a humidified atmosphere with 5% CO_2_ at 37°C.

The study was approved by the Ethics Committee of Hunan Normal University, Changsha, China.

### Medicines and chemical reagents

NOC was synthesized at the Institute of Pharmacy and Pharmacology, University of South China, as previously described (11). NOC has a molecular weight of 299 kD, appears as yellow crystals and has a purity of 99.0%. NOC was dissolved in dimethyl sulfoxide (DMSO) and was prepared as a 10 mmol/l stock solution. The antibody against Bim was purchased from Calbiochem (San Diego, CA, USA). Antibodies against phospho-Akt (Ser^473^), Akt, FOXO3a, p-FOXO3a (Thr^32^), cytochrome *c*, caspase-9 and caspase-3 were purchased from Cell Signaling Technology (Danvers, MA, USA). The antibody against β-actin was purchased from Santa Cruz Biotechnology (Santa Cruz, CA, USA) and LY294002 was from Sigma-Aldrich (St. Louis, MO, USA). Lipofectamine 2000 was purchased from Invitrogen Life Technologies. All other chemicals used were of analytical grade and were purchased from Fisher Scientific (Suwanee, GA, USA) and Sigma-Aldrich.

### Flow cytometry (FCM) analysis

To detect cell apoptotic rates, cells were seeded at a density of 4×10^6^ cells/ml in 100 ml culture flasks for 24 h and then treated for 24 h with medium containing various concentrations of ChR or NOC and 10% fetal bovine serum. Propidium iodide staining for DNA content analysis was performed, as previously described (16).

Mitochondrial membrane potential (ΔΨ_m_) was measured by FCM using cationic lipophilic green fluorochrome rhodamine-123 (Rh123; Molecular Probes, Eugene, OR, USA). Disruption of ΔΨ_m_ is associated with a lack of Rh123 retention and a decrease in fluorescence. Briefly, cells were washed twice with phosphate-buffered saline (PBS) and incubated with 1 *μ*g/ml Rh123 at 37°C for 30 min. Cells were then washed twice with PBS and Rh123 intensity was determined by FCM. Cells with reduced fluorescence (less Rh123) were counted as having lost mitochondrial membrane potential.

### Plasmids and transfections

A control non-specific siRNA (UUCUCCGAACGUGUCACGUdTdT) was purchased from Dharmacon, Inc. (Lafayette, CO, USA). FOXO3a siRNA (ACUCCGGGUCCAGCUCCAC) and Bim siRNA (5′-GATCCGT TCTGAGTGTGACCGAGA-3′) were synthesized by Shanghai GenePharma Co., Ltd. (Shanghai, China). Transfection of siRNA was carried out with Lipofectamine 2000 according to the manufacturer’s instructions. Forty-eight hours after transfection, the cells were exposed to either DMSO (control), 40 *μ*mol/l ChR or 10 *μ*mol/l NOC for 24 h. The cells were then collected and processed for western blot analysis and functional assays.

### Cellular fractionation

To measure the release of cytochrome *c*, cells were fractionated into cytosolic and mitochondrial fractions as described by Reuter *et al*(17). In brief, cells were incubated in buffer containing 20 mM HEPES-KOH (pH 7.2), 10 mM KCl, 1.5 mM MgCl_2_, 1 mM ethylenediamime tetraacetic acid (EDTA), 0.1 mM phenylmethylsulfonyl fluoride, 10 *μ*g/ml leupeptin and 10 *μ*g/ml aprotinin at 4°C for 10 min and then cells were homogenized with a Dounce homogenizer for 20 strokes. After addition of buffer containing 210 mM mannitol, 70 mM sucrose, 5 mM ethylene glycol tetraacetic acid (EGTA) and 5 mM Tris-HCl (pH 7.5), the homogenates were centrifuged at 1,000 × g for 10 min at 4°C. The supernatants were further centrifuged at 15,000 × g for 30 min at 4°C and collected as a cytosolic fraction.

### Western blot analysis

Western blot analysis was carried out as previously described (16). Cells were lysed in lysis buffer by incubating for 20 min at 4°C. Protein concentrations were determined using the Bio-Rad assay system (Bio-Rad, Hercules, CA, USA). Total proteins were fractionated using sodium dodecyl sulphate-polyacrylamide gel electrophoresis (SDS-PAGE) and transferred onto a polyvinylidene fluoride membrane (PVDF; Millipore, Bedford, MA. USA). Antibodies against phospho-Akt (Ser^473^), Akt, FOXO3a, p-FOXO3a (Thr^32^), Bim and β-actin were used as primary antibodies. The signals were detected using an enhanced chemiluminescence (ECL) advanced western blot analysis system (Amersham Pharmacia Biotech Inc., Piscataway, NJ, USA).

### Statistical analysis

A database was set up with the SPSS 15.0 software package (SPSS Inc., Chicago, IL, USA) for analysis. Data were represented as mean ± standard deviation (SD). The means of multiple groups were compared with one-way analysis of variance (ANOVA), after checking variance for equality. The comparisons among the means were performed using the least significant difference (LSD) method. Statistical comparison was also performed with a two-tailed t-test when appropriate. P<0.05 was considered to indicate a statistically significant difference.

## Results

### NOC induces apoptosis of MDA-MB-453 cells through a mitochondrial death cascade

**Figure 1:**
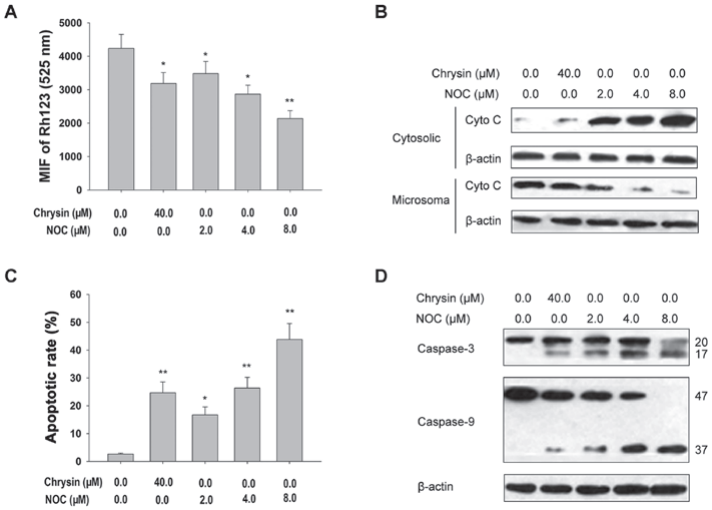
Effects of NOC on mitochondrial apoptotic events in MDA-MB-453 cells. (A) MDA-MB-453 cells were treated with the indicated concentrations of NOC or chrysin for 24 h. The mean fluorescence intensity of Rh123 was measured by FCM. Data shown are means ± SD (n=3). ^*^P<0.05; ^**^P<0.01 vs. 0.1% DMSO. (B) Cell treatment was the same as in (A). Cytochrome *c* was examined using western blotting in mitochondrial and cytoplasmic lysates and β-actin was used as the loading control. (C) Cell treatment was the same as in (A). Caspase-3 and -9 expression was determined by western blotting of the total cell lysates and β-actin was used as the loading control. (D) Cell treatment was the same as in (A). The apoptotic rate was analyzed by FCM using PI staining. Data shown are means ± SD (n=3). ^*^P<0.05; ^**^P<0.01 vs. 0.1% DMSO. NOC, 5,7-dihydroxy-8-nitrochrysin; FCM, flow cytometry; SD, standard deviation; DMSO, dimethyl sulfoxide; PI, propidium iodide.

Since NOC-induced apoptosis of breast cancer cells is associated with the production of ROS (12), we sought to determine whether NOC activates the mitochondrial death cascade by triggering apoptosis of the MDA-MB-453 cell line. We first examined the effects of NOC on several important events in the mitochondrial apoptotic pathway. Our results indicated the following: i) treatment with NOC clearly elicited ΔΨ_m_ dissipation, as demonstrated by the decrease in Rh123-derived fluorescence in FCM assays (Fig. 1A); ii) NOC triggered a rapid release of cytochrome *c* from the mitochondria to the cytoplasm, as documented by western blot analysis using cytosolic extracts (Fig. 1B); and iii) treatment with NOC caused activation of caspase-9 and -3 and increased the rate of apoptosis (Fig. 1C and D). Similar results were observed in MDA-MB-453 cells treated with ChR (Fig. 1). These data demonstrate that NOC-induced apoptosis is involved in the mitochondrial death pathway.

### NOC activates FOXO3a in MDA-MB-453 cells

**Figure 2:**
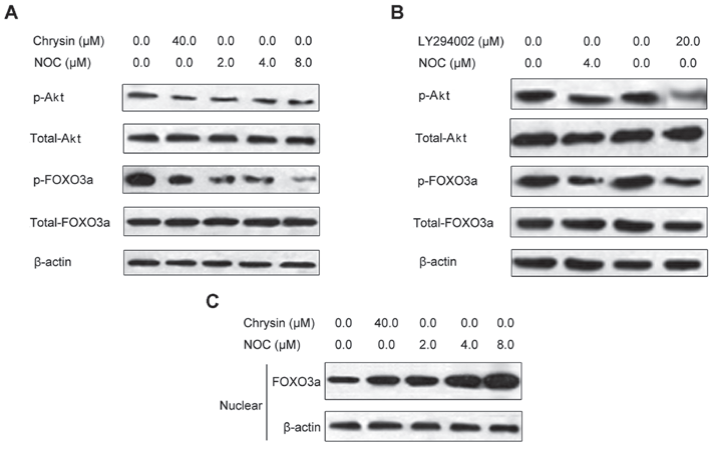
Effects of NOC on the phosphorylated protein levels of Akt and FOXO3a in MDA-MB-453 cells. (A) MDA-MB-453 cells were treated with the indicated concentrations of NOC or chrysin for 24 h. Expression of Akt and FOXO3a phosphorylated proteins were examined by western blotting of the total cell lysates and β-actin was used as the loading control. (B) Cell treatment was the same as in (A). Expression of FOXO3a protein was determined by western blotting of nuclear lysates and β-actin was used as the loading control. (C) MDA-MB-453 cells were treated with 4.0 *μ*M NOC or 20.0 *μ*M LY294002 for 24 h. Expression of Akt and FOXO3a phosphorylated proteins were examined by western blotting of the cell nuclear lysates and β-actin was used as the loading control. NOC, 7-dihydroxy-8-nitrochrysin; FOXO3a, forkhead box O3a.

We previously reported that NOC inhibited the phosphorylation of Akt in MDA-MB-453 cells (12). To further investigate whether NOC affects the expression of the downstream targets of Akt, we tested the effects of NOC on Akt and its downstream molecule FOXO3a. We observed that NOC inhibited the phosphorylation of Akt and FOXO3a in MDA-MB-453 cells (Fig. 2A). We also observed that levels of FOXO3a were increased in nuclear lysate following NOC treatment (Fig. 2B). This suggests that the increased ratio of FOXO3a to phospho-FOXO3a in the cytoplasm and nuclei of MDA-MB-453 cells represents retention of a greater amount of active FOXO3a in the nuclear compartment, thereby inducing cancer cell apoptosis. Similar results were observed in MDA-MB-453 cells treated with ChR (Fig. 2A and B). To further confirm the effects of NOC on FOXO3a, we conducted studies using LY294002, a specific phosphoinositide 3-kinase (PI3K) inhibitor. We observed that LY294002 treatment decreased phosphorylation levels of Akt and FOXO3a (Fig. 2C), similar to the effects of NOC. This suggests that the effect of NOC on FOXO3a is mediated through Akt signaling.

### FOXO3a activation is required for induction of apoptosis by NOC in MDA-MB-453 cells

**Figure 3:**
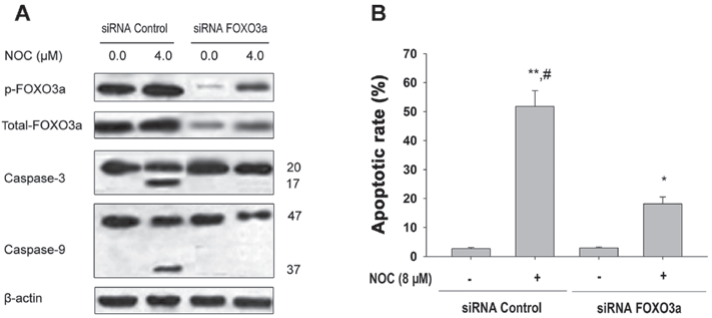
Effects of FOXO3a downregulation by siRNA transfection on FOXO3a expression and apoptosis in MDA-MB-453 cells. (A) MDA-MB-453 cells were transfected with 100 nM siRNA control or the siRNA duplexes against FOXO3a mRNA. Forty-eight hours after transfection, the cells were treated with 4.0 *μ*M NOC for 24 h. Western blotting of p-FOXO3a, FOXO3a, caspase-3 and caspase-9 was completed to confirm the downregulation of FOXO3a and the effects of caspase-3 and -9 cleavage by siRNA transfection. β-actin was used as the loading control. (B) MDA-MB-453 cells were transfected with 100 nM siRNA control or the siRNA duplexes against FOXO3a mRNA. Forty-eight hours after transfection, cells were treated with 8.0 *μ*M NOC for 24 h. The apoptotic rate was analyzed by FCM using PI staining. Data shown are means ± SD (n=3). ^*^P<0.05; ^**^P<0.01 vs. 0.1% DMSO; ^#^P<0.05 vs. the same concentration of NOC in combination with siRNA FOXO3a transfection. FOXO3a, forkhead box O3a; NOC, 7-dihydroxy-8-nitrochrysin; FCM, flow cytometry; PI, propidium iodide; SD, standard deviation; DMSO, dimethyl sulfoxide.

We next examined whether activation of FOXO3a affects NOC-induced caspase-3 activity and apoptosis. NOC induced caspase-3 activity and apoptosis in MDA-MB-453/control siRNA cells. Inhibition of FOXO3a expression by specific siRNA, significantly inhibited NOC-induced caspase-3 and-9 activity and apoptosis (Fig. 3A and B). These data suggest that NOC induces caspase-3 activity and apoptosis through activation of FOXO3a, while silencing of FOXO3a inhibits activities associated with caspase-3 and -9 and apoptosis.

### FOXO3a activation regulates expression of Bim in MDA-MB-453 cells

**Figure 4:**
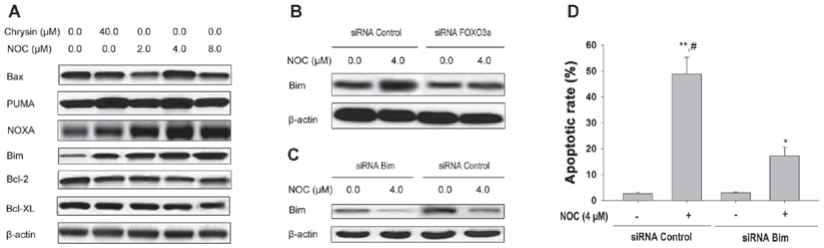
Effects of NOC on the expression of Bcl-2 family proteins in MDA-MB-453 cells. (A) MDA-MB-453 cells were treated with the indicated concentrations of NOC or chrysin for 24 h. Expressions of Bax, PUMA, Noxa, Bim, Bcl-2 and Bcl-XL proteins were examined by western blotting of total cell lysates and β-actin was used as the loading control. (B) MDA-MB-453 cells were transfected with 100 nM siRNA control or the siRNA duplexes against FOXO3a mRNA. Forty-eight hours after transfection, cells were treated with 4.0 *μ*M NOC for 24 h. Expression of Bim protein was determined by western blotting of total cell lysates and β-actin was used as the loading control. (C) MDA-MB-453 cells were transfected with 100 nM siRNA control or the siRNA duplexes against Bim mRNA. Forty-eight hours after transfection, cells were treated with 4.0 *μ*M NOC for 24 h. Expression of Bim protein was determined by western blotting of total cell lysates and β-actin was used as the loading control. (D) MDA-MB-453 cells were transfected with 100 nM siRNA control or siRNA duplexes against Bim mRNA. Forty-eight hours after transfection, the cells were treated with 4.0 *μ*M NOC for 24 h. The apoptotic rate was analyzed by FCM using PI staining. Data shown are means ± SD (n=3). ^*^P<0.05; ^**^P<0.01 vs. 0.1% DMSO; ^#^P<0.05 vs. the same concentration of NOC in combination with siRNA Bim transfection. NOC, 7-dihydroxy-8-nitrochrysin; Bcl-2, B cell lymphoma 2; Bax, Bcl-2 associated X protein; PUMA, p53 upregulated modulator of apoptosis; FCM, flow cytometry; PI, propidium iodide; SD, standard deviation; DMSO, dimethyl sulfoxide.

Mitochondrial dysfunction plays an important role in breast cancer apoptosis. Changes in the expression of B cell lymphoma (Bcl)-2-family proteins are involved in ChR-induced apoptosis of cancer cells (18). However, it is unclear whether BH3 proteins function in MDA-MB-453 cells following NOC treatment. Therefore, we investigated the expression of Bcl-2-family proteins in MDA-MB-453 cells following NOC treatment. Pro-apoptotic proteins, including Bcl-2-associated X protein (Bax), p53 upregulated modulator of apoptosis (PUMA) and Noxa, were slightly increased in MDA-MB-453 cells following NOC treatment (Fig. 4A). Anti-apoptotic Bcl-2 and Bcl-extra large (XL) proteins also exhibited a slight decrease. However, in contrast to other proteins, the expression level of Bim was markedly increased following NOC treatment, providing evidence that Bim was involved in apoptotic cell death in MDA-MB-453 cells. Similar results were observed in MDA-MB-453 cells treated with ChR (Fig. 4A).

We next examined the effect of FOXO3a activation on the expression of Bim. This gene is a direct target of the FOXO3a transcription factor. MDA-MB-453 cells were pretreated with FOXO3a siRNA followed by treatment with NOC for 24 h and the expression of Bim was measured by western blotting (Fig. 4B). FOXO3a siRNA attenuated the induction of Bim expression by NOC treatment (Fig. 4B). These data suggest that NOC may also regulate the expression of the FOXO3a transcriptional target, Bim.

We next sought to determine whether Bim is involved in NOC-induced apoptosis of MDA-MB-453 cells. Cells were transiently transfected with Bim-specific siRNA following the determination of caspase-3 activity and apoptosis by NOC treatment. As shown in Fig. 4C and D, cells receiving Bim siRNA displayed a reduced proportion of Bim-induced apoptosis, suggesting that Bim upregulation mediates MDA-MB-453 cell apoptosis induced by NOC.

## Discussion

Our previous study demonstrated that Akt inactivation, ROS generation, c-Jun N-terminal kinase (JNK) activation and caspase activation contribute to NOC-induced apoptosis in human breast cancer MDA-MB-453 cells. However, the precise molecular mechanisms responsible for the apoptotic effect of NOC remain to be fully characterized. In this study, we first identified that NOC induces apoptosis of MDA-MB-453 cells and this is primarily mediated through the mitochondrial death pathway. This study demonstrated that activation of the Akt/FOXO3a axis, followed by increased Bim expression, contributes to NOC-induced apoptosis in MDA-MB-453 cells.

Apoptosis is initiated via two alternative signaling pathways, the death receptor-mediated extrinsic apoptotic pathway and the mitochondrion-mediated intrinsic apoptotic pathway (17,19). Mitochondria play critical roles in the regulation of various apoptotic processes, including drug-induced apoptosis (20). Our results demonstrated that NOC induces MDA-MB-453 cell apoptosis by activating the mitochondrion-mediated intrinsic apoptotic pathway (Fig. 1). Bcl-2 family proteins regulate mitochondria-dependent apoptosis, with the balance of anti- and pro-apoptotic members arbitrating life or death decisions. Bim, a pro-apoptotic member of the Bcl-2 family, causes apoptosis by disrupting mitochondrial integrity. In the present study, we identified that NOC induces Bim expression and that Bim siRNA attenuates NOC-induced MDA-MB-453 cell apoptosis (Fig. 4). This suggests that Bim expression is causally related to NOC-induced MDA-MB-453 cell apoptosis. Furthermore, NOC-induced Bim expression was inhibited by FOXO3a siRNA transfection (Fig. 4). Thus, it is plausible that NOC activates FOXO3a, thereby causing Bim expression and subsequent cell apoptosis.

The FOXO3a transcription factor is a tumor suppressor that is inactivated in the majority of human cancers, owing to over-activation of the PI3K/Akt pathway (21–23). The FOXO3a protein regulates a variety of genes that affect cell proliferation, survival, metabolism and responses to stress (24). Upon the activation of PI3K/Akt signaling, FOXO3a undergoes Akt-mediated phosphorylation, which promotes binding to the 14-3-3 protein and nuclear export through CRM1 (also known as XPO1 and exportin 1) and cytoplasmic sequestration. FOXO3a proteins translocate to cell nuclei for execution of their transcriptional functions when the PI3K/Akt pathway is inhibited, including under stress conditions or in the absence of growth or survival factors (21).

This study revealed a novel mechanism utilized by NOC to induce apoptosis in MDA-MB-453 cells. Apoptotic resistance in HER-2/neu-overexpressing breast cancer cells is mediated by a loss of FOXO3a activity (25) and we demonstrated that this pathway is inhibited by NOC. Upstream, NOC activates FOXO3a by targeting the Akt pathway. Downstream, NOC induces FOXO3a activity and leads to increased expression of Bim, which induces apoptosis in breast cancer cells. This study demonstrated that NOC inhibits Akt activation, thereby preventing FOXO3a phosphorylation (inactivation) in breast cancer cells. This reveals a new mechanism of NOC-induced apoptosis of breast cancer cells.

In summary, our study demonstrated that NOC induces apoptosis in breast cancer cells by promoting FOXO3a activity. This results in the expression of Bim via inactivation of Akt. Further studies are required to assess the anticancer activity of NOC *in vivo*. A thorough understanding of the mechanisms of NOC may lead to discovery and development of novel therapeutic molecules for the treatment and prevention of human breast cancer.
